# Amyloid-β in Alzheimer’s disease – front and centre after all?

**DOI:** 10.1042/NS20220086

**Published:** 2023-01-06

**Authors:** Caroline Weglinski, Alexander Jeans

**Affiliations:** Department of Pharmacology, University of Oxford, Oxford OX1 3QT, U.K.

**Keywords:** Alzheimers disease, Amyloid beta, synapses

## Abstract

The amyloid hypothesis, which proposes that accumulation of the peptide amyloid-β at synapses is the key driver of Alzheimer’s disease (AD) pathogenesis, has been the dominant idea in the field of Alzheimer’s research for nearly 30 years. Recently, however, serious doubts about its validity have emerged, largely motivated by disappointing results from anti-amyloid therapeutics in clinical trials. As a result, much of the AD research effort has shifted to understanding the roles of a variety of other entities implicated in pathogenesis, such as microglia, astrocytes, apolipoprotein E and several others. All undoubtedly play an important role, but the nature of this has in many cases remained unclear, partly due to their pleiotropic functions. Here, we propose that all of these AD-related entities share at least one overlapping function, which is the local regulation of amyloid-β levels, and that this may be critical to their role in AD pathogenesis. We also review what is currently known of the actions of amyloid-β at the synapse in health and disease, and consider in particular how it might interact with the key AD-associated protein tau in the disease setting. There is much compelling evidence in support of the amyloid hypothesis; rather than detract from this, the implication of many disparate AD-associated cell types, molecules and processes in the regulation of amyloid-β levels may lend further support.

## The amyloid hypothesis – and the emergence of doubt

Alzheimer’s disease (AD) is a chronic neurodegenerative disease and the most common cause of dementia. Globally, the number of people living with dementia is predicted to increase from 55 million in 2020 to 78 million in 2030 and 139 million in 2050 [1]. AD causes progressive cognitive impairment and is characterized by widespread accumulation of both the amyloid-β (Aβ) peptide, particularly in its aggregated, oligomeric form, and the protein tau throughout the brain as, respectively, amyloid plaques and neurofibrillary tangles [2]. There is an acute need for treatments that stop or reverse the progression of AD as most are purely symptomatic, and only one drug currently approved for clinical use, Aducanumab, may have genuine disease-modifying activity, although even this is controversial [3]. Understanding the mechanisms of AD pathogenesis has therefore been a key aim for the research community for many years. An important landmark in this effort was the identification of mutations in genes that cause an early-onset, familial form of AD, accounting for 5–10% of all cases, although it is important to note that not all early-onset AD has this aetiology [4]. All of the responsible mutations identified affect either the genes coding for the amyloid precursor protein (APP) from which Aβ is formed via a multistep processing pathway, or the genes coding for the presenilin (PS) 1 and 2 proteins that function within this processing pathway as part of an enzyme complex responsible for cleaving Aβ from its immediate precursor [5]. These observations suggested that Aβ may be both necessary and sufficient for pathogenesis and led directly to the establishment of the amyloid hypothesis, which proposes that extracellular deposits of Aβ, particularly the longer and more aggregation-prone Aβ_1–42_ isoform, trigger a chain of events that includes the recruitment and hyperphosphorylation of tau, and leads to the loss of synapses and eventually neurons themselves that characterise AD [6]. There is substantial and compelling support for this hypothesis, ranging from the genetics of familial AD to a wealth of *in vitro* studies demonstrating a variety of neurotoxic effects of AD-associated forms of Aβ [7], and the AD field enthusiastically embraced it, to the point that it was for many years the dominant influence in setting consensus research priorities [8].

One of the key predictions of the amyloid hypothesis is of course that reducing levels of Aβ and amyloid plaques will ameliorate the symptoms of AD and slow or even arrest its progression [9]. This prospect is particularly exciting because of the current lack of any effective disease-modifying therapy. Accordingly, an enormous investment of time and resources has been directed at the development of amyloid-depleting agents that have predominantly taken the form of monoclonal antibodies directed against the peptide itself, including some specifically directed at particular pathology-associated Aβ assembly states, such as small oligomers. These have been extensively tested, and a number of them have entered clinical trials, with a number having progressed as far as stage III. The results to date have been ambivalent. Most of these agents have unfortunately proved ineffective [3], although recently an antibody directed specifically against a protofibrillar form of Aβ has been shown to have a moderate ability to slow cognitive decline [10]. While this result gives some cause for a very cautious optimism, it is true that results to date from this approach have been overall disappointing, and Aβ is clearly not as effective a therapeutic target as once hoped.

This has naturally led to intense debate within the field. Many proponents of the amyloid hypothesis have held firm in their conviction that Aβ remains a prime candidate therapeutic target in AD, and they assert that the trial failures indicate only that these agents have not engaged the target in an effective way, or perhaps not been given at the optimal point in the natural history of the disease [11]. On the other hand, there is also a large and perhaps increasing number of investigators who take the view that so many individual failures cast grave doubt on the value of Aβ as a therapeutic target in AD. Indeed, for some these results call into question the assumed critical role of Aβ in pathogenesis. Perhaps more widespread is a more conservative view that, while still acknowledging the necessity of Aβ for the development of AD, suggests that the trial failures indicate that its role lies well upstream in the pathogenic cascade, and that it is far from being the proximate cause of cognitive decline [12].

## A change in direction?

These doubts have led to a profound shift in the landscape of AD research. Where once Aβ was the prime focus, and much of the field was dedicated to understanding the biology of this peptide at all levels, from molecular signalling pathways to its effects on neuronal networks, the focus has now shifted, and instead, several other factors that have been implicated in driving AD pathogenesis in various different ways have gained prominence. Areas that have garnered particular attention are neuroinflammation and microglial function, the role of astrocytes, the major genetic risk factor *APOE4*, AD-associated hyperactivity in hippocampal and cortical neuronal networks, and sleep. In addition, the other signature protein of AD histopathology, tau, has been the subject of intensive investigation.

**Figure 1:**
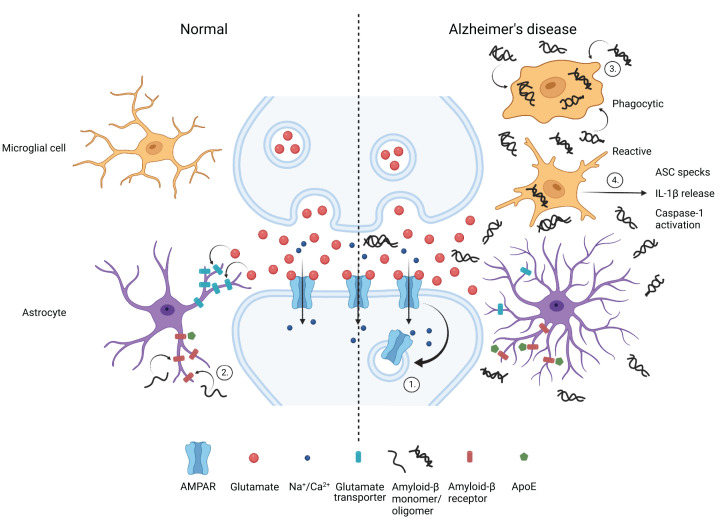
The impact of glial cells and ApoE on local Aβ concentration A schematic representation of a synapse, the left half illustrating the roles of microglia, astrocytes and the lipoprotein ligand ApoE in regulating Aβ release and degradation under physiological conditions. The right half illustrates how these processes can change in the context of AD. High concentrations of pathological (here shown as oligomeric) Aβ drive synaptic weakening, initially via AMPA receptor internalization (1). Several factors regulate local levels of the various forms of Aβ. Astrocytes serve numerous functions, including reuptake of released neurotransmitter as shown on the left of the figure. However, they also take up Aβ via scavenging receptors (2), a process that can be blocked by competitive binding of ApoE as shown on the right. Note that the AD risk associated variant ApoE4 has a particularly high affinity for Aβ scavenging receptors. In AD, microglia that have differentiated towards a phagocytic phenotype can also clear Aβ by phagocytosis (3), while a subpopulation of microglia also differentiates to a secretory phenotype with a far lower phagocytic capacity, this subtype becoming more prominent as the disease advances. These secrete a variety of pro-inflammatory mediators, including cytokines such as IL-1β and inflammasome-related multiprotein complexes known as ASC specks (4), which drive changes deleterious to neurons including the activation of caspases. Image created using Biorender.

These various entities present their own difficulties for researchers. All have multiple functional roles that together span a very broad repertoire, and in many cases it is not yet clear which function(s) are relevant to AD pathogenesis. However, it has become apparent that there is at least one area of functional overlap between all of them, and it is perhaps a surprising one given the reasons for the recent increase in attention that they have all received: all have been shown to have a strong impact on the level or activity of Aβ. We propose that this observation is potentially highly significant as it suggests that Aβ, despite the substantial concerns over the level of its involvement in AD, may very well still play a critical, central role in pathogenesis. Therefore, in this piece, we will first briefly summarise what is known of the impact of each of the entities or processes named above on Aβ. Some of these are also summarized in Figure 1. We will then summarise and re-examine some of the key findings that support a central pathogenic role for Aβ and consider some of the wider implications of this, and in particular how Aβ as a putative central player might direct the recruitment and hyperphosphorylation of the protein tau, a critical process in pathogenesis [13].

## Could key AD-related entities converge on the regulation of Aβ?

### Neuroinflammation and microglial function

Microglia are the specialized innate immune cell of the CNS and they play a significant role in the pathology of AD [14]. They are currently the subject of a great deal of attention, and some authorities have even proposed that microgliosis and neuroinflammation be recognized as a core pathological feature of AD, along with plaque and tangle deposition [15]. Microglia have been directly implicated in both neurodegeneration [16] and synapse loss, the latter via an Aβ-dependent mechanism requiring complement receptors [17]. However, they have also been clearly implicated in the local control of Aβ levels via various mechanisms. In the brains of AD patients and model animals, many microglia display an activated, pro-inflammatory phenotype (Figure 1), and are able to internalise and phagocytose Aβ [18,19]. Several studies have shown that the functional role of microglia changes with aging and disease progression, and this may be associated with changes in the transcriptome and protein expression [20,21]. The expression of triggering receptor expressed on myeloid cells 2 (TREM2) stimulates microglial survival and phagocytosis of Aβ [22], and mutations in this protein have been identified as a risk factor for AD [23]. During early stages of disease, TREM2 levels are increased as a neuroprotective process linked to Aβ clearance [20,24]. However, as AD progresses, enhanced microglial activation, in response to advanced amyloid and tau pathology, leads to the excessive release of pro-inflammatory cytokines, which promote extensive inflammation, eventually leading to widespread neurodegeneration [25,26]. These ‘toxic’ microglia also lose their Aβ-clearing capabilities, with reduced expression of various Aβ receptors and Aβ degradative enzymes [21].

### Astrocytes

Astrocytes are crucial regulators of the brain’s inflammatory response and there are increased numbers of reactive astrocytes in AD, many exhibiting cellular hypertrophy [27]. Similar to microglia, astrocytes appear to subserve a number of different functions in the AD brain and, depending on several factors including the stage of disease, they may be either protective or toxic. Astrocytes express the necessary enzymes to produce Aβ, with cellular stress promoting its secretion [28–30]. The presence of Aβ is also able to induce the release of pro-inflammatory cytokines from astrocytes (Figure 1) that can not only regulate Aβ-induced cellular toxicity but may also have effects on the scavenging of Aβ by microglia [31]. Some familial AD mutations can drive an increase in cleavage of APP and secretion of Aβ by astrocytes, significantly contributing to the amyloid load [32]. Astrocytes also express various proteases involved in cleaving Aβ and participate in the uptake and degradation of Aβ [33]; however, the beneficial effects of activated astrocytes degrading Aβ are likely to be at least partly opposed by the neurotoxic properties of this glial cell population [34].

### *APOE* genotype

Apolipoprotein E (ApoE) is mainly produced by astrocytes in the CNS and is involved in cholesterol transport to neurons [35]. The *APOE* gene has three alleles, with *APOE4* being the major genetic risk factor for developing sporadic AD [36]. ApoE4 promotes AD risk via multiple mechanisms that include diverse cellular processes and systems, amongst them mitochondrial function, glucose metabolism, neuroinflammation and synaptic transmission [37], as well as cerebrovascular function [38]. ApoE is also an important regulator of Aβ levels. ApoE apolipoproteins bind directly to Aβ, and the E4 variant is associated with decreased Aβ clearance and increasing seeding activity [39–41]. Mechanistically, ApoE appears to regulate clearance of Aβ by competitively binding to low density lipoprotein receptor-related protein 1 (LRP1), a surface receptor on astrocytes (Figure 1) and microglia, preventing Aβ uptake [42,43]. Confirming the role of ApoE4 in driving the accumulation of Aβ, *APOE4* transgenic mice demonstrate higher Aβ levels together with enhanced Aβ deposition [44]. A similar ApoE/LRP1-dependent mechanism appears to regulate the uptake of extracellular tau, which is thought to play a role in the pathogenesis of tauopathies including AD [45].

### Neuronal network activity

Many studies have demonstrated that hyperactivity in cortico-hippocampal neuronal networks is one of the earliest changes observed in several transgenic mouse models of AD [46]. It is also found in human patients at early stages of the disease, as well as those diagnosed with mild cognitive impairment, which is often considered a prodromal stage to full-blown AD [47]. This appears to be Aβ-dependent, as neuronal hyperactivity may be acutely induced by application of exogenous soluble Aβ oligomers into the brains of wild-type mice [48]. It has also been shown that spontaneous epileptic activity, associated with network hypersynchrony, can be observed in EEG recordings from transgenic mice models with high Aβ levels, as a result of an imbalance between excitation and inhibition in both local as well as large-scale brain networks [49]. Studies have identified the deficits in inhibitory interneurons as one potential mechanism of network hyperactivity in early AD pathogenesis [49]. It is still unclear what role, if any, these changes in neuronal activity play in pathogenesis. However, they are likely to have an indirect but significant effect on Aβ concentration since synaptic activity is a direct regulator of Aβ release, which occurs via synaptic vesicle exocytosis [50]. Indeed, it has been shown in AD model mice that regional neuronal activity is commensurate with local Aβ concentration and with subsequent amyloid plaque deposition [51], indicating that it is highly likely that enhanced activity contributes to pathogenesis via the elevation of Aβ levels in the brain, potentially along with other mechanisms.

It is relevant to note here that imaging studies in patients have provided evidence that network-level abnormalities can occur years or even decades in advance of clinical symptoms [52], so there is a long latent period in which enhanced synaptic activity could be driving the release and accumulation of Aβ. This could therefore represent an appealing extended potential window for therapeutic intervention with the aim of preventing disease development.

### Sleep

Sleep disturbances have been associated with the development of AD pathology and constitute a risk factor for the disease, although the mechanism underlying the interaction is still unclear [53]. Sleep deprivation leads to an increased concentration of pathogenic Aβ in CSF and may be linked to an increased expression of pro-inflammatory cytokines and microglial activation [54,55]. Studies have suggested that the increase in Aβ levels may be due to decreased glymphatic clearance during wakefulness [56]. Additionally, high levels of synaptic activity stimulate the release of Aβ from neurons [35].

### Tau

Tau is undoubtedly a critical effector in AD. Data from mouse models suggest that it is absolutely required in order for amyloid to trigger or initiate pathogenesis [57,58] whether this is true in the setting of human disease. In its hyperphosphorylated, disease-associated form, tau appears to be directly toxic to neuronal physiology [59]. However, it is perhaps the exception to the theme suggested above, in that tau does not itself appear to modulate Aβ levels or activity. This is in no way incompatible with a central role for Aβ in pathogenesis, as it is likely explained by the relationship between Aβ and tau simply being inverse to its relationship with the factors described above, i.e. Aβ regulates and recruits tau, rather than being regulated by it. This is specifically proposed by the amyloid hypothesis and suggests that tau is a more proximal effector of toxicity than Aβ, which would be in keeping with the observation that tau pathology correlates better with cognitive status than amyloid pathology in AD patients [60]. Although for many years the mechanism by which Aβ recruits tau to the disease process has remained a significant knowledge gap in the AD field [61], a recent study from our laboratory has identified a candidate mechanism which will be discussed later in this review.

Given the remarkable convergence of the key AD-related entities currently under investigation on the regulation of Aβ levels, notwithstanding the exception of tau which has a fundamentally different relationship with Aβ, it may be timely to review some of the major candidate mechanisms via which it could exert its pathological effects at the synapse, before briefly describing new findings that may help to address one of the weaknesses of the amyloid hypothesis, the absence of a mechanism for the downstream recruitment of tau. Firstly, however, we will give context to all of this by summarizing what we currently know of the physiological roles of Aβ and tau at the synapse.

## Physiological roles of Aβ and tau at the synapse

While Aβ and tau are best known as the central proteins involved in AD pathogenesis, they also have important roles at synapses that support normal physiological function [62]. The enzymes responsible for generating Aβ are present in the synaptic compartment, in keeping with a role for the peptide in synaptic function. The concentration of Aβ is positively regulated by neuronal activity [50], and what are considered to be physiological (picomolar) concentrations of Aβ have been shown to enhance both the probability of neurotransmitter release (an index of the efficacy of action potentials in driving vesicular release from the presynaptic terminal) and long-term potentiation (LTP), an activity-dependent process of synaptic strengthening that is thought to underlie learning and memory formation [63–65]. Indeed, APP knockout [66] and BACE1 (a critical enzyme for Aβ production) knockout mouse models show impaired LTP and cognitive deficits [67]. *In vivo*, the administration of an anti-Aβ antibody prevents short-term memory formation in a contextual fear conditioning paradigm while the infusion of picomolar amounts of Aβ was able to rescue contextual fear memory [64]. Aβ-derived peptides can also promote the survival of primary neurons [68] while, conversely, the absence of Aβ appears to be detrimental: hippocampal neurons from APP knockout mice show reduced synapse formation [69], aberrant synaptic network organization and a reduction in synaptic proteins [70,71].

Tau is also present at the synapse and is involved in a number of synaptic functions via its role in the regulation of microtubule stability and axonal transport [62]. Tau was long thought to be primarily an axonal protein [72], but it is now clear that it is also present in the postsynaptic compartment and interacts with F-actin in dendritic spines [73]. Like Aβ, tau can also be released from cells, likely via synaptic vesicle exocytosis, under physiological conditions [74]. Studies in which tau is depleted or knocked out have produced diverse results, with some reporting reduced spine density, LTP deficits and spatial memory impairments [75,76], while others have found deficits in long-term depression (LTD), an activity-dependent process of synaptic weakening, and no change in LTP [77]. Plasticity effects may be explained by indirect interactions of tau with NMDA receptors, which regulate both LTP and LTD. These interactions are mediated via tau binding to the postsynaptic scaffold protein PSD-95 [78,79] as well as to the NMDA receptor kinase fyn [80,81]. Synaptic tau seems to be particularly important for the proper function of the extrasynaptic subpopulation of NMDA receptors [82], which not only play a role in plasticity induction [83] but may also facilitate excitotoxicity [82]. Tau also modulates neuronal excitability, as tau reduction has been shown to diminish hyperexcitability in a mouse with seizure activity [84]. It also plays a role in neuron and synaptic development, since tau knockdown affects dendritic spine density [76] and causes deficits in hippocampal neurogenesis and neuronal maturation in primary neurons [85,86]. Interestingly, the application of brain-derived neurotrophic factor (BDNF) increases tau expression along with spine development, and the suppression of tau blocks the neuronal response to BDNF [87]. There is, therefore, a substantial body of work supporting the importance of the two signature proteins of AD in maintaining critical neuronal physiological functions, in particular various forms of synaptic plasticity. This may be an important consideration when designing therapeutic approaches which rely on lowering levels of either, a confound that could potentially be avoided by targeting only pathological forms of these proteins, such as oligomers or aggregates, rather than physiological ones, which are usually monomeric. This approach has already been taken to develop oligomer-selective amyloid-lowering agents [3].

## Mechanisms of synaptic dysfunction in AD

Synaptic changes are some of the earliest observed in AD and relevant models, with evidence of synaptic dysfunction appearing before the development of plaques or overt neurodegeneration [88]. Furthermore, synapse loss, which also occurs in advance of neuronal loss, is well established as the best pathological correlate of cognitive decline in AD [89]. These observations suggest that synaptic changes are central to the pathogenesis of AD, but how can pathological forms of Aβ bring these changes about?

There is evidence that Aβ may exert direct toxic effects on synapses by binding to specific receptor sites on the surface of neurons, and numerous candidates for receptors mediating this function have been proposed. These are principally surface proteins of various classes such as prion protein (PrP), metabotropic glutamate receptor 5 (mGluR5), nicotinic acetylcholine receptors (nAChR), adrenergic receptors and plasticity-associated proteins such as PirB/LilrB2 [7]. However, candidates are not limited to proteins and include membrane lipids, via which the function of surface proteins may be indirectly modulated [90]. For many of these candidates, downstream signalling pathways that mediate the pathological effects have also been proposed. Despite this abundance of receptor candidates, none has so far been convincingly shown to be both necessary and sufficient to account for all aspects of Aβ toxicity [91] Accordingly, the AD field still tends to regard the identity of the Aβ receptor(s) as an open question.

By contrast, it is widely agreed that the effect of pathological forms of Aβ on glutamatergic synapses, however, it is initiated, is to depress postsynaptic function [88]. Most of the available evidence suggests this occurs via the pathological induction of LTD or a similar process [92,93], although there is less agreement on the pathways upstream of this. Some studies have suggested that this synaptic weakening is driven by a partial blockade of NMDA receptors by Aβ oligomers [93], while others suggest that elevated extracellular glutamate may be responsible, activating extra-synaptic NMDA receptors and/or metabotropic glutamate receptors (mGluRs) that recruit LTD signalling cascades [94,95]. Elevated extracellular glutamate due to failure of reuptake has also been implicated in the genesis of the neuronal hyperactivity that is believed to contribute to circuit dysfunction in early AD [96]. Finally, there is evidence that Aβ can induce an LTD-like process via activation of a pathological PrP-mGluR5 signalling platform [97]. Regardless of the mechanism, it appears that Aβ-induced synaptic weakening represents the initial stage of a pathway that ultimately leads to synapse loss [92,93].

Other mechanisms of Aβ-mediated toxicity have also been proposed, including dysregulation of neuronal intracellular Ca^2+^ homeostasis, which constitutes another potential route to synaptic failure and dendritic spine loss [98]. The acute application of Aβ can increase neuronal resting Ca^2+^ levels, an effect which is primarily mediated by postsynaptic NMDA receptors [99]. It is also possible that intracellular Ca^2+^ stores contribute to the dyshomeostasis and increased Ca^2+^ levels either via calcium-induced calcium release (CICR), or via a deficit in their Ca^2+^ buffering function [100,101]. Elevated intracellular Ca^2+^ can lead to the activation of calcineurin, in turn driving downstream neuronal dysfunction and synapse loss [102].

The extent to which some or all of the mechanisms of Aβ toxicity described above may require the involvement of tau is not entirely clear, but it is certainly the case that pathological Aβ appears able (in line with the amyloid hypothesis) to recruit tau, which becomes hyperphosphorylated and aggregated thereby eliciting toxic effects [13]. This is discussed in more detail below. The mechanisms of downstream tau toxicity remain incompletely understood; however, studies using genetic tauopathy models, while not always directly relevant to AD, have highlighted some significant effects. Since tau is involved physiologically in microtubule stability and intracellular transport, it appears that pathological alterations in tau function cause critical disruptions in protein trafficking to the synapse [103–105]. Mitochondrial transport to presynaptic terminals is essential for efficient synaptic vesicle release since mitochondria regulate Ca^2+^ and ATP levels [106], and pathological tau is also able to bind kinesin and compete with microtubule cargo [107], preventing the synaptic transport of mitochondria which can lead to synaptic damage and eventual loss [108]. Finally, pathological tau may be mislocalized to presynaptic terminals, where it can directly bind to synaptic vesicles, impairing exocytosis [109].

## Intersection of amyloid and tau – dysfunction at the synapse

The amyloid hypothesis predicts that pathological changes in the level or assembly state of Aβ precede, and are responsible for, the recruitment of tau [6]. Indeed, there is good evidence to support this idea, in particular the observation that Aβ accumulation as a result of familial AD mutations in APP and related genes leads to abundant tau deposition, while mutations in tau alone produce no subsequent development of Aβ pathology [110–112]. Studies conducted in neuronal cultures using exogenous applications of pathological Aβ show that this treatment can directly induce tau hyperphosphorylation and cause neuritic degeneration [113], and it has also been shown in mouse models of AD that the presence of tau is necessary for canonical Aβ-induced disease phenotypes such as loss of LTP [58] and cognitive decline [57] to develop. More generally, a number of studies show an interplay or synergy between Aβ and tau in pathogenesis; some of these show that Aβ has a facilitatory effect on the development or spread of tau pathology [114,115], while others demonstrate that tau may be required for, or at least potentiate, certain mechanisms of Aβ-induced synaptic degeneration [116,117].

Aβ causes missorting of tau, which is normally predominantly axonal, to dendritic spines, resulting in increased activity of tau-interacting kinases, an increase in local Ca^2+^ concentration and gradual spine loss [118,119]. Although there is no evidence of direct interaction between Aβ and tau, the proteins may indirectly interact via common links to different kinases involved in cell signalling pathways [120]. One potential example of this is the plasticity-associated tyrosine kinase fyn, which can be activated by Aβ [121], and which binds to tau in dendritic spines; here Aβ can drive fyn-mediated phosphorylation and functionally potentiation of NMDA receptors, leading to excitotoxicity and neuronal death [122]. Accordingly, some toxic effects of Aβ can be ameliorated by removing tau, which both disrupts the postsynaptic targeting of fyn and decreases NMDA receptor phosphorylation [122].

As might be expected, Aβ and tau can also co-operate to bring about network-level as well as synaptic impairments [120]. One recent study has provided evidence that Aβ and tau alone each have opposing effects on the activity of neuronal circuits – Aβ causes hyperactivity, while soluble pathogenic tau suppresses activity. However, when both are present together, neuronal activity is silenced suggesting that the effect of tau is dominant [123]. This study appears to contradict previous findings, which show that tau is essential for Aβ-induced hyperexcitability [57,124]. The discrepancy might be explained by the use of different forms of tau, with the effects documented in the latter studies being reliant only on the presence (or absence) of endogenous tau, while the Aβ/tau transgene co-expression study overexpressed a familial tauopathy-associated form of mutant tau.

**Figure 2:**
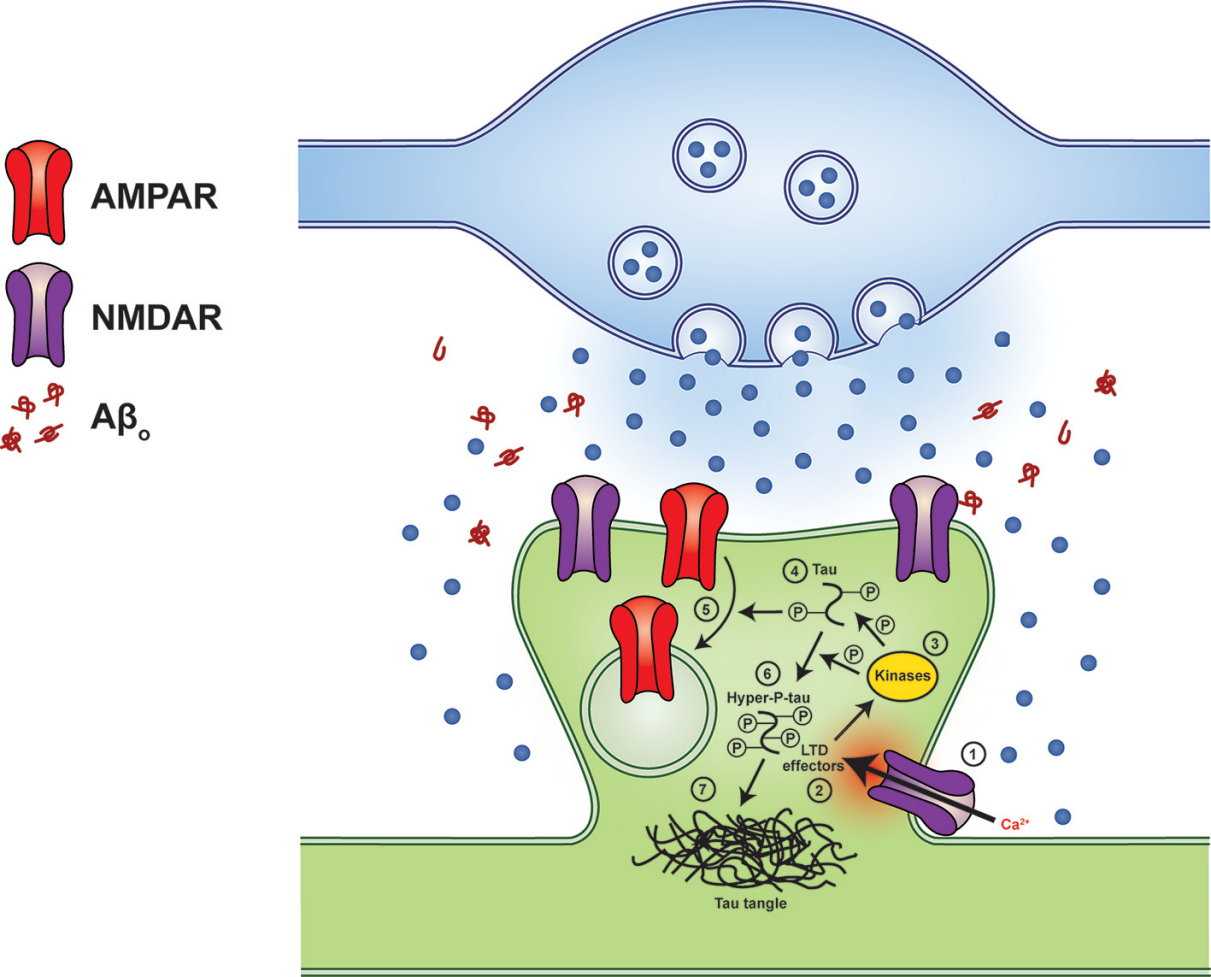
Proposed mechanism of Aβ-induced tau hyperphosphorylation A detailed proposed mechanism of Aβ-induced tau hyperphosphorylation based on data from Taylor et al. [129], from which this figure is also adapted. Oligomeric Aβ enhances the probability of neurotransmitter release from the presynaptic terminal, resulting in increased low-frequency synaptic activity and/or activation of extrasynaptic NMDAR (1), thus promoting the induction of NMDAR-dependent LTD. LTD is initiated by an NMDAR-dependent Ca^2+^ influx that activates a variety of LTD effector proteins (2) that in turn activate LTD-associated kinases (3). During the physiological induction of LTD, these kinases phosphorylate tau (4), which alters its affinity for microtubules and helps to promote endocytosis and internalization of synaptic AMPAR (5). If the LTD induction stimulus is excessive or unusually prolonged, due to pathologically increased synaptic activity, kinase activation may be inappropriately sustained, potentially leading to hyperphosphorylation of tau at non-physiological residues (6). Hyperphosphorylated tau is in itself toxic, and will eventually form stable aggregates that give rise to the histopathological inclusions (dystrophic neurites and neurofibrillary tangles) that are diagnostic of AD (7).

The specific mechanism by which Aβ might interact with or recruit tau has been a long-standing open question in the AD field. Recently, however, a study from our laboratory has identified a candidate mechanism that could help to fill this key knowledge gap. Based on the observations described above that LTD, or at least an LTD-like process, seems to be induced during Aβ-induced synaptic toxicity, and on data from a recent study showing that, under physiological conditions, LTD induction phosphorylates tau albeit at just two residues [125], we hypothesized that the pathological LTD induced by Aβ could drive tau phosphorylation, and potentially at residues in addition to those described for physiological LTD. Using pharmacological and optogenetic approaches in cultured brain slices, we confirmed that repeated administration of an NMDAR-dependent low frequency stimulation LTD induction protocol that is based on one used in physiological plasticity studies (but delivered in excess) indeed drives phosphorylation of endogenous tau at pathology-associated residues. We further implicated Aβ-induced increases in presynaptic probability of neurotransmitter release, which have been reported by us and others previously [126–128] in generating the low-frequency synaptic activity required to induce LTD [129]. This study provides evidence for a direct mechanistic link between the two key proteins of AD (Figure 2) and suggests that LTD-associated proteins might constitute potential therapeutic targets.

We should, however, also acknowledge that some studies have disputed the idea of a linear model in which tau pathology is purely a downstream effect of Aβ, and instead propose that there may be a reciprocal interaction between Aβ and tau [130,131]. Overexpressing human tau in an AD mouse model with APP/PS1 mutations led to an increase in plaque size but no change in synapse loss when compared with APP/PS1 mice lacking the tau transgene [131]. In another study, the deletion of tau significantly decreased levels of soluble and insoluble Aβ in APP/PS1 mutant mice, likely via an impairment of β-secretase cleavage of APP due to a disruption in BACE1 trafficking [132]. It is clear that there is still much to uncover concerning the interplay of Aβ and tau, and this will remain an important area of study for many years to come.

## Future prospects and outstanding questions

Despite this existing body of knowledge, many unanswered questions remain in the AD field, and addressing these constructively will be necessary in order to identify the novel therapeutic targets that are so badly needed. Despite a recent clinical trial of one agent showing a moderate effect on slowing cognitive decline [10], most trials of amyloid-lowering therapies have ended in failure [133] and it does appear that Aβ may not be an optimal target for therapeutic intervention. Accordingly, focus has shifted to, amongst other things, the development of anti-tau therapies, which have shown promise in pre-clinical studies [134]. If tau does indeed lie downstream of Aβ in the pathological cascade, targeting it may be more beneficial during the symptomatic phase of disease; however, these interventions must balance the advantageous effects of disrupting pathology with the harmful effects of reducing normal physiological tau function [62]. Particularly in view of a possible synergistic relationship between Aβ and tau, a combinatorial approach aimed at reducing both may be optimal [120].

Transgenic mouse models of AD have considerably advanced our understanding of the disease, although there has been a lack of reproducibility in some findings. This is likely to be partly due to background genetic differences between mice [135], but there is also the potential confounding factor of artefacts arising from supraphysiological levels of transgene expression, which was a feature of most of the early AD mouse models owing to the use of non-endogenous promoters [135]. Currently, there is an increasing trend towards the use of more representative ‘knock-in’ mouse models, in which the endogenous mouse *app* gene is replaced by a humanized version bearing one or more familial AD mutations, expressed under control of the endogenous *app* promoter to ensure these animals are free of transgene overexpression [136]. Additional mutations in certain genes, such as *TREM2* and *APOE4*, linked to sporadic AD, have also been introduced in several mouse models [137,138]. Inducible mutations in AD-related genes also have a role to play in understanding AD pathogenesis, as they allow important questions to be asked via control of the chronology, as well as the level, of transgene expression [139].

One key open question that we have, intentionally, avoided addressing here is the role of different assembly states of Aβ (principally small oligomers versus larger species) in both normal neuronal physiology and AD pathogenesis. A similar question exists regarding tau. The answers are still unclear, and studying different assembly states of these proteins specifically represents a great experimental challenge. Aβ belongs to a class known as intrinsically disordered proteins (IDP), which lack a stable tertiary structure in physiological conditions, and the conformation of Aβ is highly dependent on its environment. Furthermore, since different assembly states exist in equilibrium with one another, any ‘pure’ preparation of one species will not remain so for long as the equilibrium is constantly being re-established [7]. A further outstanding question is the role of intracellular vs. extracellular Aβ and tau. Although tau is traditionally considered to act intracellularly, a fraction appears to be secreted via exosomes [140] and removal of this seems beneficial in certain mouse models [141–143]. Conversely, Aβ is usually studied as a secreted, extracellular peptide but it is also found within neurons and there is some evidence that this intracellular fraction plays a significant role in impairments in synaptic function and plasticity [144].

## Data Availability

No data were generated for this review

## Open Access

Open access for this article was enabled by the participation of University of Oxford in an all-inclusive *Read & Publish* agreement with Portland Press and the Biochemical Society under a transformative agreement with JISC.

## Abbreviations

Aβ: amyloid-β
AD: Alzheimer’s disease
AMPAR: α-amino-3-hydroxy-5-methyl-4-isoxazolepropionic acid receptor
ApoE: apolipoprotein E
APP: amyloid precursor protein
ASC: apoptosis-associated speck-like protein containing a CARD
ATP: adenosine triphosphate
BACE1: β-site APP cleaving enzyme 1
BDNF: brain-derived neurotrophic factor
CICR: calcium-induced calcium release
CNS: central nervous system
CSF: cerebrospinal fluid
EEG: electroencephalogram
IDP: intrinsically disordered protein
IL-1β: interleukin-1β
LRP1: low-density lipoprotein receptor-related protein 1
LTD: long-term depression
LTP: long-term potentiation
mGluR: metabotropic glutamate receptor
nAChR: nicotinic acetylcholine receptor
NMDAR: N-methyl-D-aspartate receptor
PirB/LilrB2: paired immunoglobulin-like receptor B/leukocyte immunoglobulin-like receptor B2
PrP: prion protein
PS: presenilin
PSD-95: postsynaptic density protein of 95 kilodaltons
TREM2: triggering receptor expressed on myeloid cells 2
